# The protective effects of Ramelteon against 6‐OHDA‐induced cellular senescence in human SH‐SY5Y neuronal cells

**DOI:** 10.1002/brb3.2278

**Published:** 2021-07-23

**Authors:** Dan Liu, Xinyi Gu, Fang Han, Ming Cai, Weijie Liu, Li Han, Qiang Ma

**Affiliations:** ^1^ Department of Neurology The Affiliated ZhongShan Hospital of Dalian University Dalian China; ^2^ Department of Imaging The Affiliated ZhongShan Hospital of Dalian University Dalian China

**Keywords:** 6‐OHDA, cell senescence, Nrf2, oxidative stress, Parkinson's disease, Ramelteon

## Abstract

**Background and purpose:**

Parkinson's disease (PD) is a severe neurodegenerative disease with high morbidity in the elderly population. 6‐OHDA‐induced cell senescence is reported to be involved in the pathogenesis of PD. Ramelteon is an oral hypnotic agent that specifically targets the receptors of the suprachiasmatic nucleus in the human hypothalamus. Here, an investigation is made to see whether Ramelteon possesses a beneficial effect against 6‐OHDA‐induced cellular senescence in human SH‐SY5Y neuronal cells.

**Methods:**

The release of LDH was detected to assess cytotoxicity and flow cytometry was conducted to evaluate the cell cycle. The telomerase activity and the SA‐β‐Gal assay were performed to determine the state of cell senescence. Oxidative stress was evaluated by detecting the release of H_2_O_2_. The expressions of p21, p53, and Nrf2 were measured using the qRT‐PCR and Western blotting assay. siRNA technology was used to knock down the expression level of Nrf2 in SH‐SY5Y neuronal cells.

**Results:**

First, it was found that Ramelteon mitigated cell cycle arrest in the G0/G1 phase in 6‐OHDA‐challenged SH‐SY5Y neuronal cells. Second, treatment with Ramelteon alleviated cellular senescence in 6‐OHDA‐treated SH‐SY5Y neuronal cells by increasing telomerase activity and reducing the activity of SA‐β‐gal. It was also found that Ramelteon reduced the expressions of p21 and p53. Notably, Ramelteon attenuated 6‐OHDA‐induced oxidative stress by increasing the expression of Nrf2. Silencing of Nrf2 abolished the protective effects of Ramelteon against 6‐OHDA‐induced cellular senescence. Based on these findings, it was concluded that Ramelteon alleviated 6‐OHDA‐induced cellular senescence by increasing the expression of Nrf2 in human SH‐SY5Y neuronal cells.

**Conclusion:**

Ramelteon protected against 6‐OHDA‐induced cellular senescence in human SH‐SY5Y neuronal cells through activating the Nrf2 signaling pathway.

## 1 INTRODUCTION

Parkinson's disease (PD) is a neurodegenerative disease that is characterized by the loss of dopaminergic neurons and the formation of Lewy bodies. Bradykinesia, static tremor, myotonia, and postural and gait abnormalities are the classic motor symptoms of PD and are possibly accompanied by non‐motor symptoms such as constipation, loss of smell, anxiety, depression, cognitive impairment, and dementia (Beitz, 2014; Opara et al., 2017). The global morbidity of PD is approximately 10–50 patients in a 100,000 population, annually (Pringsheim et al., 2014; Tysnes & Storstein, 2017; von Campenhausen et al., 2005). It is predicted that with age, the total number of PD patients would be doubled by 2030 (Dorsey et al., 2007). At present, the pathological mechanism of PD is still unknown. The main strategy for the treatment of PD includes increasing the concentration of dopamine and direct stimulation of dopamine receptors, but this can only ameliorate the PD symptoms, rather than suppressing the process of PD. Therefore, it is important to explore the pathogenesis for the treatment of clinical PD.

Currently, several animal and cellular models have been proven to effectively simulate the pathogenesis of PD (Duty & Jenner, 2011), including 6‐OHDA (Blum et al., 2001). Although the pathogenesis of 6‐OHDA‐induced neurotoxicity remains unclear, it is supposed that oxidative stress (Gomez‐Lazaro, Galindo, et al., 2008), cell death mediated by mitochondria (Gomez‐Lazaro, Bonekamp, et al., 2008), and cell senescence (Mei et al., 2015) are the possible elements involved in the pathological mechanism of 6‐OHDA. In fact, it is reported that cell senescence is an important factor that contributes to the pathogenesis of neurodegenerative diseases, including PD (Hou et al., 2019). Carmen claimed that cell senescence could be taken as a clinical marker for predicting the progression in PD (Martin‐Ruiz et al., 2020). Dong reported that the upregulation of the p53/p21 signaling pathway contributes to cellular senescence and the accumulation of α‐synuclein, which is an important pathological feature of PD (Ho et al., 2019). The pathological mechanism of dopaminergic neuron degeneration is closely related to the accumulation of reactive oxygen species (ROS), which induces the activation of oxidative stress (Dias et al., 2013). It is critical for maintaining the regular function of redox‐sensitive signaling protein in neuronal cells and the survival of neurons to maintain the homeostasis of redox (Apel & Hirt, 2004; Chinta & Andersen, 2008). Excessively released ROS are mainly located in the mitochondria of neurons and astrocytes and induce a series of pathological processes, including neuroinflammation, degradation of dopamine, mitochondrial dysfunction, aging, and exhaustion of glutathione (GSH), which finally contribute to the development of PD (Meiser et al., 2013). Fao et al. (2019) claimed the significance of the Nrf2/ARE signaling pathway, an important regulatory pathway for oxidative stress, in the development of PD. Therefore, targeting cellular senescence and oxidative stress might be an effective method for the treatment of PD.

Ramelteon is an oral hypnotic agent developed by Takeda and approved by the US Food and Drug Administration in 2005. It is the first melatonin receptor agonist for clinical treatment of insomnia (Hatta et al., 2014; Kuriyama et al., 2014). Ramelteon regulates the periodic biorhythm within 24 h by specifically targeting the receptors of the suprachiasmatic nucleus of the human hypothalamus (Rawashdeh et al., 2011). Recently, it has been reported that Ramelteon relieves oxidative stress by activating the Nrf2 signaling pathway, thereby protecting against traumatic brain injury (J. Wang et al., 2019). In this study, the protective effect of Ramelteon on 6‐OHDA‐treated SH‐SY5Y neuronal cells, as well as the underlying mechanism, will be investigated to explore the potential therapeutic property of Ramelteon on PD.

## 2 MATERIALS AND METHODS

### 2.1 Cell culture and treatments

Human SH‐SY5Y neuronal cells were purchased from Shanghai Kanglang Biological Technology Co., Ltd (Shanghai, China) and cultured in F12 + Dulbecco's modified Eagle's medium (DMEM) containing 10% fetal bovine serum (FBS, Invitrogen, MA, USA), 50 U/ml penicillin, and 50 μg/ml streptomycin solution (Sigma‐Aldrich, USA). Cells were cultured in a cell incubator at 37°C and 5% CO_2_. Cells were treated with 6‐OHDA (#H4381, purity > 97%, Sigma‐Aldrich, USA) (50 μM) (Chandrasekhar et al., 2018) with or without 30 or 60 nM Ramelteon (#SML2262, purity > 98%, Sigma‐Aldrich, USA) (Nishiyama & Hirai, 2014) for 24 h.

### 2.2 LDH release

The cell viability of the treated SH‐SY5Y neuronal cells was evaluated by detecting the release of LDH. After incubating with 1% Triton X‐100 for 45 min, the samples were incubated with the culture medium supplemented with lactate, NAD+, diaphorase, 0.004% BSA, 0.15% sucrose, and 2‐p‐iodophenyl‐3‐nitrophenyl tetrazolium chloride (INT) in the dark. Subsequently, the absorbance at 490 nm was detected with a microplate reader (Bio‐Rad, California, USA).

### 2.3 Cell cycle assay

After washing the treated SH‐SY5Y neuronal cells with the PBS buffer, the cells were fixed with 70% ethanol at 4°C overnight. Subsequently, the cells were stained with propidium iodide (PI) (Sigma, Missouri, USA) and RNase A (Sigma, Missouri, USA) for 30 min. Lastly, the cell cycle of treated SH‐SY5Y neuronal cells was analyzed with the BD Flow Cytometer System (BD, New Jersey, USA) and the data were analyzed utilizing the CellQuest Pro software (BD, New Jersey, USA).

### 2.4 Measurement of intracellular H_2_O_2_

Cells were seeded in a 96‐well cell culture plate and treated with 6‐OHDA (50 μM) with or without 30 or 60 nM Ramelteon for 24 h. After that, intracellular ROS was measured using a commercial kit (#K204‐200, Biovision, USA).

### 2.5 Telomerase activity

The treated SH‐SY5Y neuronal cells were lysed with CHAPS buffer, and centrifugated at 15,000 × g for 30 min. The concentration of the total proteins in the cells was quantified using the BCA kit (Beyotime, Shanghai, China), followed by measuring the telomerase activity utilizing the TeloTAGGG Telomerase PCR ELISA Plus Kit (Roche, Basel, Switzerland). Lastly, the samples and each primer were added into the TRAP‐PCR reaction system, followed by quantification using RT‐PCR assay.

### 2.6 β‐galactosidase (SA‐β‐gal) staining

In brief, the treated SH‐SY5Y neuronal cells were washed with PBS buffer and then fixed with 2% paraformaldehyde at room temperature for 5 min. Subsequently, the cells were incubated with fresh SA‐β‐gal stain solution at 37°C. Lastly, after being photographed, the percentage of SA‐β‐gal positive perinuclear blue cells was recorded.

### 2.7 Real‐time PCR analysis

The RNA extraction kit (Takara, Tokyo, China) was used to isolate the total RNA from the treated SH‐SY5Y neuronal cells following the instructions of the manufacturer. Subsequently, a NanoDrop spectrophotometer (Thermo, MA, USA) was used to quantify the concentration of RNA and specific RT primers were used to transcribe the RNA into cDNA. The RNA reaction procedure was conducted with the SYBR Premix Ex Taq TM (Takara, Tokyo, China) and an Applied Bio‐Rad CFX96 Sequence Detection system (Bio‐Rad, California, USA), followed by determining the relative expression level of target genes by the threshold cycle (Ct) using the 2^−ΔΔCt^ method after normalization with the expression level of GAPDH. The following primers were used:

human p53, F: 5′‐GAAGACCCAGGTCCAGATGA‐3′, R:  5′‐CTCCGTCATGTGCTG TGACT‐3′;

human p21, F:5′‐ACCCATGCGGCAGCAA‐3′, R: 5′‐GCCATTAGCGCATCACA‐3′;

human GAPDH, F: 5′‐GGAGCGAGATCCCTCCAAAAT‐3′, R: 5′‐GGCTGTTGTCATACTTCTCATGG‐3′.

### 2.8 Western blotting assay

The nuclear and cytoplasmic protein extraction kit (Beyotime, Shanghai, China) was used to extract the total protein from the treated SH‐SY5Y neuronal cells, and then quantified with a BCA kit (Beyotime, Shanghai, China). Approximately 40 μg of protein was loaded and separated with a 12% SDS‐polyacrylamide gel (SDS‐PAGE) and then further transferred to the PVDF membrane (Millipore, MIT, USA). After blocking the non‐specific proteins using 5% non‐fat milk, the membrane was incubated with primary antibodies against p53 (1:2000, #2524, CST, Boston, USA), p21 (1:3000, #2947, CST, Boston, USA), Nrf2 (1:1500, #12721, CST, Boston, USA), and β‐actin (1:10000, #4970, CST, Boston, USA) at 4 °C overnight, followed by being incubated with anti‐mouse IgG, HRP‐linked antibody (1:2000, #7076, CST, Boston, USA), and anti‐rabbit IgG, HRP‐linked antibody (1:3000, #7074, CST, Boston, USA) at room temperature for 2 h. Lastly, the blots were incubated with the ECL reagents (Beyotime, Shanghai, China) and exposed to Tanon 5200‐multi (Tanon, Shanghai, China). The bands of the blot were scanned, selected, and the sum optical density was quantified using the Kodak Digital Science 1D software (Eastman Kodak Company, USA). Data were exported for statistical analysis and normalized to β‐actin.

### 2.9 Statistical analysis

Data were obtained from at least three independent experiments and expressed as mean ± standard deviations. Statistical analysis was performed with analysis of variance (ANOVA), followed by Tukey's post hoc test using the SPSS software (SPSS 19.0; SPSS, Inc., Chicago, IL, USA). *p* < .05 was considered to indicate a statistically significant difference.

## 3 RESULTS

### 3.1 The effects of Ramelteon on cytotoxicity of human SH‐SY5Y neuronal cells

**FIGURE 1:**
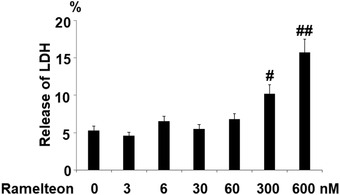
The effects of Ramelteon on cytotoxicity of human SH‐SY5Y neuronal cells. (a) Molecular structure of Ramelteon. (b) Cells were stimulated with 3, 6, 30, 60, 300, and 600 nM for 24 h. Release of LDH was measured (#, ##, *p* < .05, .01 vs. control group).

In order to screen the optimized concentration of Ramelteon, the SH‐SY5Y neuronal cells were stimulated with 3, 6, 30, 60, 300, and 600 nM for 24 h, followed by evaluating the LDH release. As shown in Figure 1, as the concentration of Ramelteon increased from 3 to 60 nM, no significant difference was observed on the release of LDH. However, the release of LDH was significantly elevated to 10.2% and 15.7% by stimulation with 300, and 600 nM Ramelteon. Therefore, 30, and 60 nM Ramelteon were used in the subsequent experiments.

### 3.2 Ramelteon mitigated cell cycle arrest in G0/G1 phase in 6‐OHDA‐treated SH‐SY5Y neuronal cells

**FIGURE 2:**
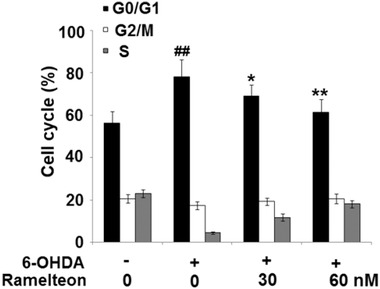
Ramelteon mitigated cell cycle arrest in G0/G1 phase in 6‐OHDA‐treated SH‐SY5Y neuronal cells. Cells were treated with 6‐OHDA (50 μM) with or without 30 or 60 nM Ramelteon for 24 h. Cell cycle was assayed (##*p* < .01 vs. control, *, **, *p* <.05, .01 vs. 6‐OHDA).

To evaluate the effects of Ramelteon on the cell cycle, cells were treated with 6‐OHDA (50 μM) with or without 30 or 60 nM Ramelteon for 24 h. As shown in Figure 2, compared to control, the percentage of cells arrested in the G0/G1 phase was significantly elevated in the 6‐OHDA group, but greatly suppressed by the treatment with Ramelteon in a dose‐dependent manner.

### 3.3 Ramelteon alleviated cellular senescence in 6‐OHDA‐treated SH‐SY5Y neuronal cells

**FIGURE 3:**
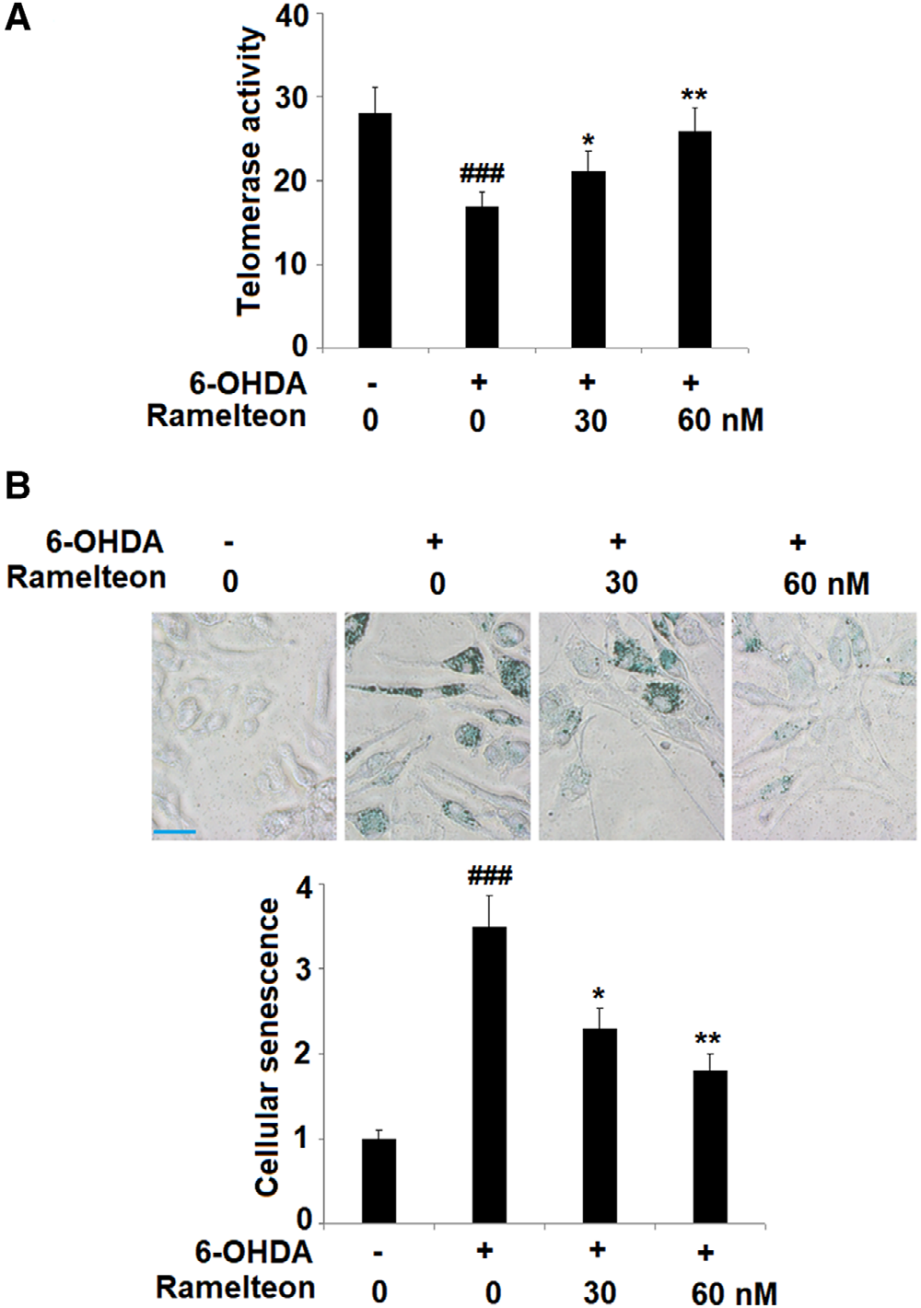
Ramelteon alleviated 6‐OHDA‐induced cellular senescence in 6‐OHDA‐treated SH‐SY5Y neuronal cells. Cells were treated with 6‐OHDA (50 μM) with or without 30 or 60 nM Ramelteon for 24 h. (a) Telomerase activity. (b) Cellular senescence was measured using SA‐β‐Gal staining. Scale bar, 50 μm (###, *p* < .001 vs. control, *, **, *p* < .05, .01 vs. 6‐OHDA)

We further evaluated the state of cell senescence by detecting the telomerase activity and the activity of SA‐β‐Gal. As shown in Figure 3a, compared to the control, the telomerase activity was decreased from 28.1 to 16.9 IU/L by stimulation with 6‐OHDA and significantly elevated to 21.2 and 25.9 IU/L by the introduction of 30, and 60 nM Ramelteon. In addition, compared to the control, the activity of SA‐β‐gal (Figure 3b) was dramatically promoted by stimulation with 6‐OHDA but greatly suppressed by treatment with Ramelteon in a dose‐dependent manner.

### 3.4 Ramelteon reduced the 6‐OHDA‐induced expressions of p53 and p21 in 6‐OHDA‐treated SH‐SY5Y neuronal cells

**FIGURE 4:**
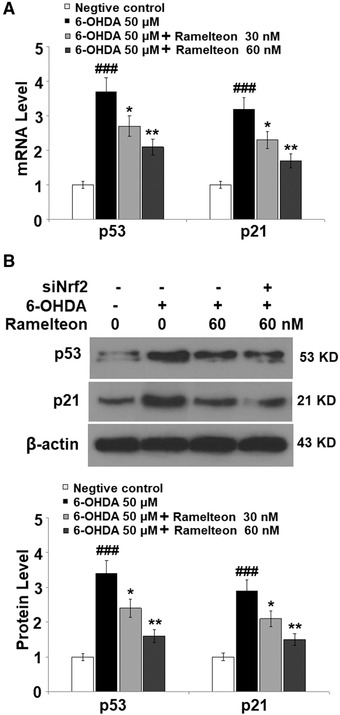
Ramelteon reduced 6‐OHDA‐induced expressions of p53 and p21 in 6‐OHDA‐treated SH‐SY5Y neuronal cells. Cells were treated with 6‐OHDA (50 μM) with or without 30 or 60 nM Ramelteon for 24 h. (a) mRNA of p53 and p21. (b) Protein of p53 and p21 (###, *p* < .001 vs. control, *, **, *p* <.05, .01 vs. 6‐OHDA)

We further explored the potential mechanism underlying the regulatory effect of Ramelteon on cell senescence. As shown in Figure 4, the expression levels of p21 and p53 were significantly elevated by stimulation with 6‐OHDA and pronouncedly reversed by the administration of Ramelteon. These data indicate that Ramelteon might alleviate the state of cell senescence in SH‐SY5Y neuronal cells induced by 6‐OHDA through inhibiting the p21/p53 signaling pathway.

### 3.5 Ramelteon attenuated 6‐OHDA‐induced oxidative stress by increasing the expression of Nrf2

**FIGURE 5:**
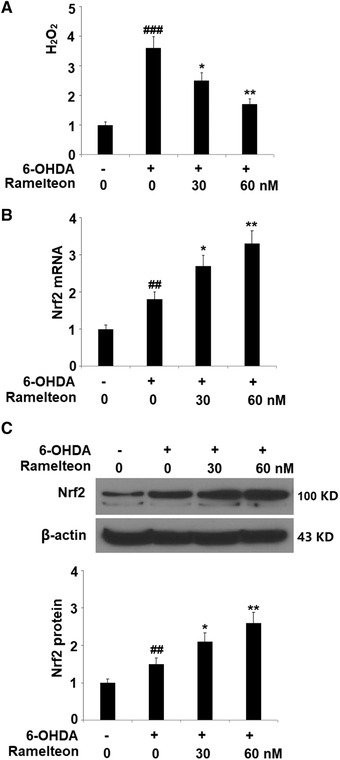
Ramelteon attenuated 6‐OHDA‐induced oxidative stress by increasing the expression of Nrf2. Cells were treated with 6‐OHDA (50 μM) with or without 30 or 60 nM Ramelteon for 24 h. (a) Production of H_2_O_2_ was measured using a commercial kit. (b) mRNA levels of Nrf2. (c) Protein level of Nrf2 (##, ###, *p* < .01, .001 vs. control, *, **, *p* < .05, .01 vs. 6‐OHDA)

To determine the effect of Ramelteon on oxidative stress, the production of H_2_O_2_ in the treated SH‐SY5Y neuronal cells was detected. As shown in Figure 5a, the secretion of H_2_O_2_ was significantly elevated by stimulation with 6‐OHDA, and suppressed by the introduction of Ramelteon. In addition, the downregulated Nrf2 (Figure 5b) in 6‐OHDA‐treated SH‐SY5Y neuronal cells was pronouncedly upregulated by treatment with Ramelteon. These data indicate that the 6‐OHDA‐induced oxidative stress in SH‐SY5Y neuronal cells was significantly ameliorated by Ramelteon.

### 3.6 Silencing of Nrf2 abolished the protective effects of Ramelteon against 6‐OHDA‐induced cellular senescence

**FIGURE 6:**
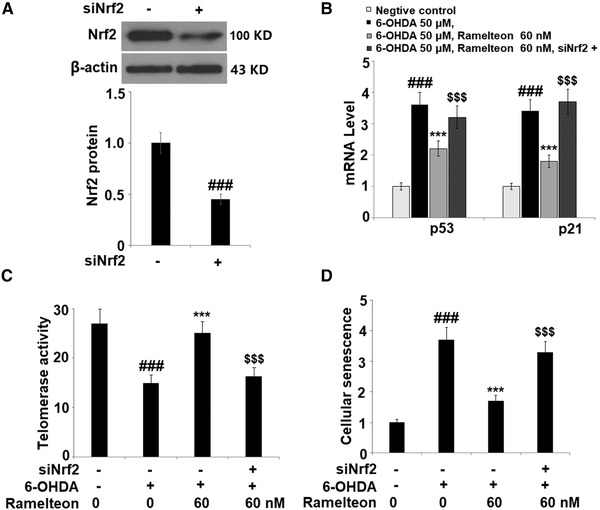
Silencing of Nrf2 abolished the protective effects of Ramelteon against 6‐OHDA‐induced cellular senescence. Cells were transfected with siRNA Nrf2, followed by stimulation with 6‐OHDA (50 μM) with or without 60 nM Ramelteon for 24 h. (a) Western blot analysis revealed successful knockdown of Nrf2. (b) mRNA of p53 and p21. (c) Telomerase activity. (d) Cellular senescence was measured using SA‐β‐Gal staining (###, *p* < .001 vs. vehicle group, ***, *p* < .001 vs. 6‐OHDA, $$$, *p* < .001 vs. 6‐OHDA+ Ramelteon group)

To verify the mechanism underlying the protective effects of Ramelteon on cell senescence, cells were transfected with siRNA Nrf2, followed by stimulation with 6‐OHDA (50 μM) with or without 60 nM Ramelteon for 24 h. As shown in Figure 6a, the expression level of Nrf2 was significantly suppressed in siRNA transfected cells, indicating that the Nrf2 knockdown in the SH‐SY5Y neuronal cells was successfully established. The elevated expression levels of p21 and p53 were significantly suppressed by the introduction of Ramelteon and later reversed by the knockdown of Nrf2 (Figure 6b). Importantly, compared to the 6‐OHDA group, the telomerase activity was elevated from 15.3 to 25.2 IU/L by treatment with Ramelteon and then reversed to 14.2 IU/L following the knockdown of Nrf2 (Figure 6c). In addition, the increased activity of SA‐β‐Gal by stimulation with 6‐OHDA was dramatically suppressed by Ramelteon, and further elevated by the knockdown of Nrf2 (Figure 6d). These data indicate that the protective effects of Ramelteon against 6‐OHDA‐induced cellular senescence were significantly abolished by the silencing of Nrf2.

## 4 DISCUSSION

Currently, the permanent dopamine depletion model induced by neurotoxin is commonly used in in‐vitro experiments to simulate the pathogenesis of PD, including 6‐hydroxydopamine (6‐OHDA), tetrahydropyridine (MPTP), and methylamphetamine (MA) (Dauer & Przedborski, 2003). 6‐OHDA is a hydroxylated neurotransmitter analog of dopamine and is decomposed into hydroxyl radical and quinones materials, which contributes to the degeneration of dopamine neurons and the reduction of dopamine function in the substantia nigra striatum. As a consequence, PD‐like symptoms are developed (Simola et al., 2007). The production of H_2_O_2_ and semiquinone are induced following the oxidation of 6‐OHDA. The released H_2_O_2_ activates the caspase‐3/7 signaling pathway by inducing oxidative stress to trigger the release of cytochrome C, finally contributing to the apoptosis of neurons (Hanrott et al., 2006). Semiquinone activates caspase‐3 to induce apoptosis through the caspase‐8 and caspase‐12 signaling pathways (Saito et al., 2007). In the present study, 6‐OHDA was used to simulate PD‐like symptoms in human SH‐SY5Y neuronal cells, which was verified by the arrested cell cycle and elevated release of H_2_O_2_. Through treatment with Ramelteon, we found that the arrested cell cycle was significantly alleviated and the release of H_2_O_2_ was vividly inhibited, indicating a potential protective effect of Ramelteon on neurotoxin‐induced injury in neurons. In our future work, the apoptotic state and the activity of the caspase‐3 signaling pathway will be evaluated to further confirm the protective effect of Ramelteon on neurotoxin‐induced toxicity in neurons.

Oxidative stress is mainly induced by the excessive release and accumulation of ROS, which is widely regarded as the internal cause of cell senescence (Z. Wang et al., 2013). Under a normal physiological state, the production and elimination of ROS are balanced by the oxidation and anti‐oxidation system. However, due to external stimulation, excessive release of ROS is induced and the accumulated ROS cannot be eliminated by the anti‐oxidation system, which ultimately leads to the development of oxidative stress (Tonnies & Trushina, 2017). DNA damages, including DNA double strands breakage, are triggered by the activation of oxidative stress. Under an oxidative stress state, the terminal of DNA damage is unlocked by the protein complex and the injury signal is provided by the produced signal strand DNA, resulting in the activation of ataxia telangiectasia mutated (ATM) kinase. Subsequently, the phosphorylation of H2A around damaged DNA is induced and the p21/p53 signaling pathway is activated, which finally contributes to the development of cell senescence (Rai et al., 2009). In the present study, we found that the p21/p53 signaling pathway was significantly activated by stimulation with 6‐OHDA, accompanied by the symptoms of cell senescence, which was reversed by the introduction of Ramelteon, indicating that the 6‐OHDA‐induced cell senescence in neurons could be dramatically alleviated by Ramelteon. The Nrf2 signaling pathway is an important anti‐oxidative pathway that regulates the processing of oxidative stress (Bellezza et al., 2018). The complex of Nrf2‐Keap1 is decomposed and the released Nrf2 is transferred into the nucleus when the oxidative stress is activated. The expression of heme oxygenase (HO)−1 is upregulated by the transferred Nrf2, finally contributing to the activation of the anti‐oxidation system (Loboda et al., 2016). We also found that the expression of Nrf2 was significantly elevated by treatment with Ramelteon, and the protective effects of Ramelteon against 6‐OHDA‐induced cellular senescence were significantly abolished by the silencing of Nrf2, indicating that the inhibitory effect of Ramelteon on oxidative stress was related to the activation of the Nrf2 signaling pathway. In our future work, the direct target of Ramelteon in regulating the expression of Nrf2 will be investigated to better understand the regulatory effect of Ramelteon on neurotoxin‐induced oxidative stress in neurons. In addition, an animal model of PD will be established by the administration of 6‐OHDA to verify the therapeutic property of Ramelteon against PD‐like symptoms, which will provide more evidence for the application of Ramelteon for the treatment of clinical PD.

Taken together, our data illustrate the protective effects of Ramelteon against 6‐OHDA‐induced cellular senescence in human SH‐SY5Y neuronal cells.

## Data Availability

All data included in this study are available upon request through contact with the corresponding author.
